# The Southern Ocean Exchange: porous boundaries between humpback whale breeding populations in southern polar waters

**DOI:** 10.1038/s41598-021-02612-5

**Published:** 2021-12-08

**Authors:** M. C. C. Marcondes, T. Cheeseman, J. A. Jackson, A. S. Friedlaender, L. Pallin, M. Olio, L. L. Wedekin, F. G. Daura-Jorge, J. Cardoso, J. D. F. Santos, R. C. Fortes, M. F. Araújo, M. Bassoi, V. Beaver, A. Bombosch, C. W. Clark, J. Denkinger, A. Boyle, K. Rasmussen, O. Savenko, I. C. Avila, D. M. Palacios, A. S. Kennedy, R. S. Sousa-Lima

**Affiliations:** 1 Instituto Baleia Jubarte, Caravelas, BA Brazil; 2 Happywhale, Santa Cruz, CA USA; 3 Southern Cross University, Lismore, NSW Australia; 4 British Antarctic Survey, Cambridge, UK; 5 Institute for Marine Sciences, University of California Santa Cruz, Santa Cruz, CA USA; 6 Ecology and Evolutionary Biology, University of California Santa Cruz, Santa Cruz, CA USA; 7 Socioambiental Consultores Associados, Florianópolis, SC Brazil; 8 Department of Ecology and Zoology, Universidade Federal de Santa Catarina, Florianópolis, SC Brazil; 9 PROBAV, Projeto Baleia à Vista, Ilhabela, SP Brazil; 10 Coronado Sailboat, Caravelas, BA Brazil; 11 Graduate Program in Psychobiology, Biosciences Center, Universidade Federal do Rio Grande do Norte, Natal, RN Brazil; 12 Grand Circle Cruise Line, Boston, MA USA; 13 The Polar Citizen Science Collective, The Gables, Cockermouth, Cumbria, UK; 14 Cornell Lab of Ornithology, Center for Conservation Bioacoustics, Cornell University, Ithaca, NY USA; 15 Colegio de Ciencias Biologicas y Ambientales, Universidad San Francisco de Quito, Quito, Ecuador; 16 Quark Expeditions, Toronto, Canada; 17 Panacetacea, Saint Paul, MN USA; 18 National Antarctic Scientific Center of Ukraine, Kyiv, Ukraine; 19 Ukrainian Scientific Center of Ecology of the Sea, Odessa, Ukraine; 20 Grupo de Ecología Animal, Biology Department, Universidad del Valle, Cali, Colombia; 21 Department of Fisheries, Wildlife, and Conservation Sciences, Marine Mammal Institute, Oregon State University, Newport, OR USA; 22 Cooperative Institute for Climate, Ocean, and Ecosystem Studies (CICOES), University of Washington, Seattle, WA USA; 23 Department of Physiology and Behavior, Biosciences Center, Universidade Federal do Rio Grande do Norte, Natal, RN Brazil

**Keywords:** Animal migration, Behavioural ecology, Biogeography, Biooceanography, Climate-change ecology, Conservation biology, Ecological epidemiology, Evolutionary ecology, Population dynamics

## Abstract

Humpback whales (*Megaptera novaeangliae*) are a cosmopolitan species and perform long annual migrations between low-latitude breeding areas and high-latitude feeding areas. Their breeding populations appear to be spatially and genetically segregated due to long-term, maternally inherited fidelity to natal breeding areas. In the Southern Hemisphere, some humpback whale breeding populations mix in Southern Ocean waters in summer, but very little movement between Pacific and Atlantic waters has been identified to date, suggesting these waters constituted an oceanic boundary between genetically distinct populations. Here, we present new evidence of summer co-occurrence in the West Antarctic Peninsula feeding area of two recovering humpback whale breeding populations from the Atlantic (Brazil) and Pacific (Central and South America). As humpback whale populations recover, observations like this point to the need to revise our perceptions of boundaries between stocks, particularly on high latitude feeding grounds. We suggest that this “Southern Ocean Exchange” may become more frequent as populations recover from commercial whaling and climate change modifies environmental dynamics and humpback whale prey availability.

## Introduction

### Humpback whale migration patterns and breeding stocks

Humpback whales (*Megaptera novaeangliae*) are a cosmopolitan species^1^ which can migrate up to 8500 km between seasonal breeding and feeding areas^2,3^, except for the non-migratory Arabian Sea breeding population^4^. In the Pacific and Atlantic Oceans, the southern distribution of some populations from the Northern Hemisphere overlaps in equatorial regions with the northern distribution of some populations from the Southern Hemisphere^2,3,5^. Despite these overlaps in distribution, these Northern Hemisphere and Southern Hemisphere populations are unlikely to co-occur in their breeding areas, because the seasons and directions of their northward and southward migrations segregate populations spatiotemporally^6–8^. High genetic differentiation of populations between the hemispheres suggest divergence possibly to the level of distinct subspecies^9^.

Humpback whale populations in the same hemisphere are roughly synchronized in the timing of their migration to and from feeding areas. In the ocean basins of the Southern Hemisphere, humpback breeding stocks show significant genetic population structure despite an absence of geographic barriers to dispersal^10–13^. This is promoted by maternally-directed site-fidelity where calves accompany mothers for approximately a year from natal breeding areas to the feeding areas and back^14^, and probably also by social facilitation, given growing evidence that behavior is culturally acquired in humpback whales^15,16^. Seven low-latitude breeding stocks (A to G) and six high-latitude feeding areas off the Antarctic continent (Areas I to VI) are recognized based on site fidelity and spatial segregation of catches by the International Whaling Commission (IWC) in the Southern Hemisphere^17^. Here we use the term breeding stock and breeding population interchangeably, defining them as a condition where all the individuals in an area are part of the same reproductive process, forming a self-contained unit, with emigration/immigration rates far lower than the intrinsic rate of population growth^18^.

**Figure 1:**
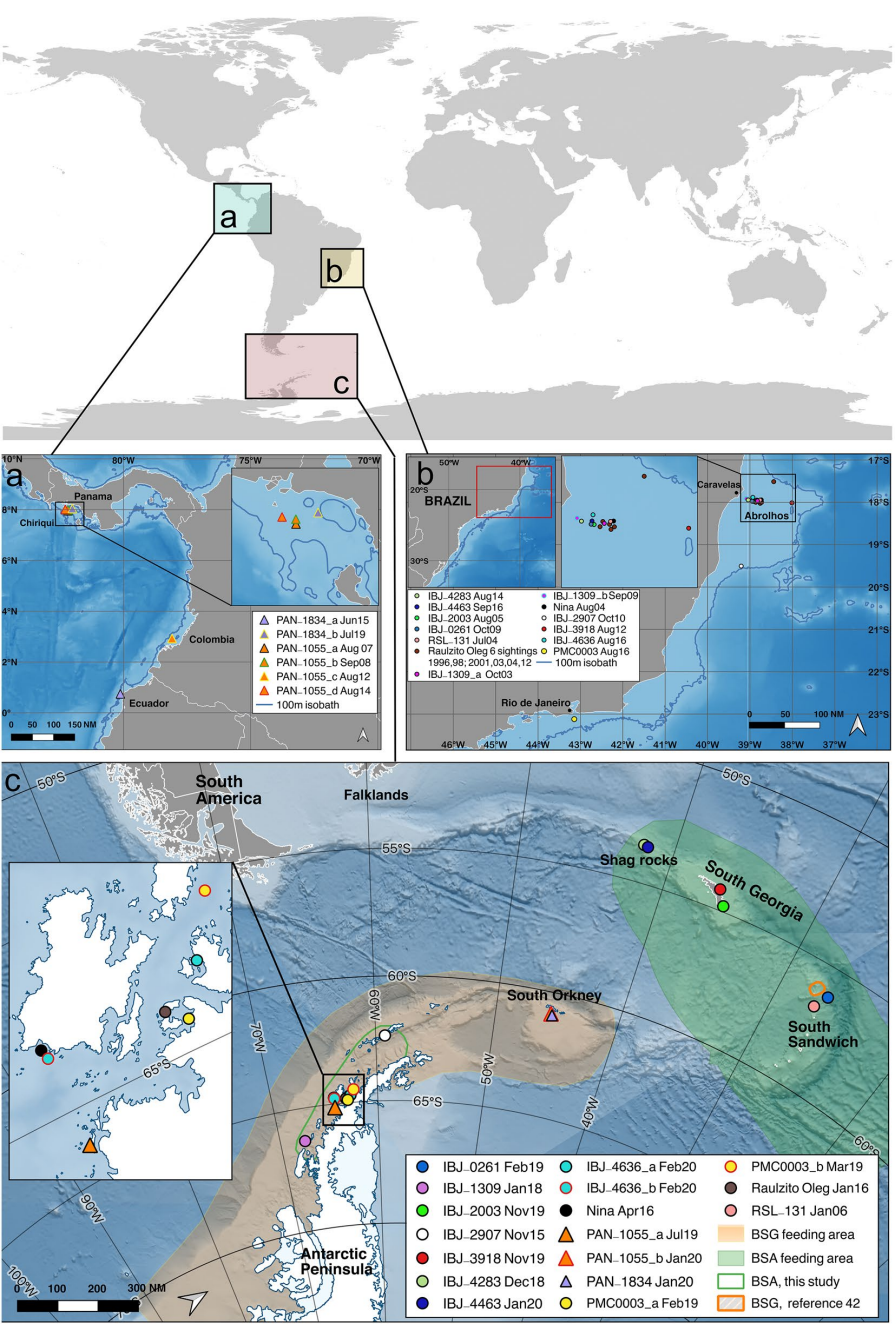
Maps of the breeding areas of breeding stock ‘G’ (BSG in **a**) and breeding stock ‘A’ (BSA in **b**), and feeding areas off the Antarctic continent (**c**). Putative feeding grounds for BSA are shown on the bottom map (**c**) in a shade of green^25–29^ and for BSG in a shade of orange^24^. Colored symbols (circle for whales from BSA and triangles for whales from BSG) indicate where individuals were photo-identified. The green line within the BSG putative feeding area delimits where BSA whales are feeding in the WAP (this study) and the orange line indicates previous information of a BSG whale feeding on the putative BSA feeding area^42^. Maps were created using QGIS version 3.16 (2021, Open Source Geospatial Foundation Project, http://qgis.osgeo.org) and Inkscape version 0.92.5 (2020, Inkscape Project, https://inkscape.org).

The breeding area of humpback whales from the breeding stock ‘G’ (BSG) is situated in the tropical and subtropical waters along the Pacific coast of Central and South America from where they seasonally migrate to the Western Antarctic Peninsula (WAP) to feed ^2,3,19,20^. Some individuals from this population do not reach the Antarctic and instead feed around the Fueguian Archipelago of Southern Chile^21^. On the Atlantic coast of South America, breeding stock ‘A’ (BSA) breeds off the coast of Brazil, from the north to the southeast, especially on the Abrolhos Bank^22,23^. The longitudinal boundary between BSG and BSA feeding areas is believed to be at or around 40° W^24^ (Fig. 1c).

Evidence provided by genetics, satellite-tracking, and photo-identification shows that BSA whales mainly feed near South Georgia and the South Sandwich Islands^25–29^ (Antarctic Area II, Fig. 1c). Satellite tracking and gravitational coordinates (local gravity accelerations associated with latitude and bed rock density) recently showed that BSA whales appear to have a persistent migratory fidelity to a corridor between Brazil and this specific sector of the South Atlantic and that those apparently “fixed” migratory trajectories do not seem to vary with changing oceanographic and geomagnetic conditions^30^. The feeding area for BSA extends west, at least to Shag Rocks (42º W; Fig. 1c) 240 km west of South Georgia^31^, and has been suggested to extend as far east as 10–20° W^28^, where stocks from West Africa (BSB) and BSA are believed to co-occur in space and time during summer feeding^32^.

### Connectivity among breeding stocks in the Southern Hemisphere

Analyses of mitochondrial DNA (mtDNA) and microsatellite loci from whales feeding in nearshore waters of the WAP determined that the natal areas of most whales are situated off Colombia (BSG) with > 94% of samples assigned to this breeding area, a few are associated with breeding grounds off French Polynesia and Samoa (breeding stock ‘F’), yet no samples were genetically assigned to Brazil (BSA)^20^. Analysis of population connectivity between Colombia, Brazil, and the WAP using microsatellite loci also showed strong restrictions to gene flow between Brazil (BSA) and Colombia (BSG), with only ~ 1–2 animals estimated to migrate between the two breeding stocks each year^33^. In a broader comparison of all Southern Hemisphere breeding stocks, the level of maternally inherited gene flow was also lowest between breeding stocks A and G, reflecting strong and significant mtDNA differentiation between the two sites^12^ and hinting that there may be long-term, natural barriers to oceanic mixing between the Atlantic and Pacific breeding stocks.

Photo-identification efforts in the WAP feeding area (between 1997 and 2003, 375 individuals photo-identified) resulted in no matches to whales in the Brazilian breeding area (between 1989 and 2000, 983 individuals photo-identified)^34^. The photo-identification catalogue built with images from the WAP is large and goes back to 1986. Satellite tracking of humpback whales in the WAP has shown some movement into the South Atlantic, but no further east than 50° W to date^35^. Nonetheless, an Ecuadorian whale has been photo-identified and re-sighted in the South Orkney Islands (45–46° W), consistent with the hypothesis that the BSG feeding range boundary may be in the South Orkney islands region^24^. Humpback whale sightings and historical catches also suggest a hiatus in distribution between the South Orkneys region and the South Atlantic feeding areas in the northern and eastern Scotia Arc^36^. Altogether, these catches, sightings, individual movements, and molecular evidence suggest that some level of segregation occurs not only between the breeding areas but also between the feeding areas associated with BSA and BSG^33,37^.

Despite the genetic and photo-identification patterns suggesting low population connectivity between oceans, photo-identified sightings of individual animals show that humpbacks can move between breeding grounds and migrate into different oceans from their natal breeding areas. Some examples of such breeding area interchange among populations in different ocean basins include: (1) a male identified by skin biopsy samples who transited from the Indian Ocean to the South Atlantic Ocean^38^, (2) a photo-identified female who moved from Brazil (BSA) to Madagascar (BSC)^39^, (3) a female photographed off Ecuador (BSG) who was later photo-identified off Brazil^40^ and most recently (4) a whale photographed off Ecuador (BSG) who was later photo-identified off Brazil^41^. Previous photo-identifications also show that whales from BSG can feed in the same areas as whales from BSA; a single whale from BSG has been photographed both in Ecuador (2131 individuals photo-identified) and the South Sandwich Islands (36° W, 23 individuals photo-identified)^42^. These cases involve movements by individuals either between different Southern Hemisphere breeding areas, or into adjacent feeding areas that are believed to be used by a different population.

The most recent case noted above was identified using a user-friendly platform that allows citizens to upload whale photographs and have their records compared to others from a large database—Happywhale^43^. Happywhale identified an opportunity to quickly expand data coverage globally by citizen science. Democratizing science through public participation and engagement brings benefits that include low cost data acquisition and increased collaboration network^44,45^ while promoting universal and equitable access to scientific data and information^46^. Here we further explore the Happywhale database to investigate where individuals co-occur at high latitudes in the Southern Hemisphere between the genetically distinct breeding stocks, mainly if individuals from Brazil (BSA) are occuring in the assumed feeding area of whales from the Pacific Central and South America (BSG).

## Results

**Figure 2:**
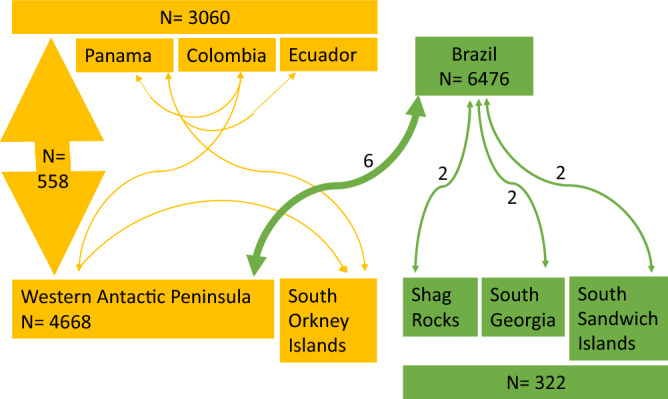
Summary of the matching results for individuals from breeding stock ‘A’ (BSA, in green) and breeding stock ‘G’ (BSG, in orange). N indicates the number of individual whales in the Happywhale catalogue from each region (N for Brazil is likely overestimated because all catalogues have not yet been cross-validated). The thickness of the arrows indicate the number of matched individuals (excepting the arrow linking the west coasts of Central and South America to the Western Antarctic Peninsula, not to scale because it is much greater, N = 558). Two particular BSG humpback whales are shown moving between the WAP all the way to the edge of the putative feeding area for the Central and South American Pacific breeding population. The number of matched individuals is noted if greater than one by each arrow connecting breeding and feeding areas.

Here we present evidence from fourteen individuals from Brazil (BSA, n = 12) and Pacific Central and South America (BSG, n = 2) matched to putative feeding areas (Table 1, Figs. 1, 2) of BSG (Fig. 1c, showed in a shade of orange) and BSA (Fig. 1c showed in a shade of green). Happywhale’s current dataset includes 4302 identified humpback whales from the WAP. Of these, 558 match to the west coasts of Central and South America (BSG), 12 match to Oceania (BSF) (T. Cheeseman, unpublished data), and six to Brazil (BSA; this study, Fig. 1 and Table 1). Two individuals from Brazil were matched to Shag Rocks, two to South Georgia, and two to the South Sandwich Islands, which are all known feeding areas for Brazilian whales, confirming the putative feeding area for the Brazilian population. But six other matches from Brazil were made with the WAP, which is the feeding area attributed to whales breeding in Pacific Central and South America (Figs. 1, 2). Additionally, two whales from Pacific Central and South America were matched at the eastern edge of the putative feeding area for BSG (South Orkney Islands). One of these two individuals (PAN-1055) was first sighted in 2007 in Central America (Panama, BSG), five years later was sighted in Colombia (Gorgona Island, BSG), matched to the WAP (Hovgaard Island) feeding area during the austral winter of 2019 and then, less than a year later, was found again in Antarctica off Signy Island, South Orkney Islands. This individual moved across the known range of BSG feeding areas within six months and may not have migrated during this time to low latitude waters (identified in Antarctica in July 2019 and in January 2020).

**Table 1:** Photographic records of individual humpback whales from breeding stock ‘A’ (BSA in green) and breeding stock ‘G’ (BSG in orange).

Whale ID	Records on breeding areas	Records on feeding areas	Δt	Additional information	Happywhale link
IBJ-4283			4 years 4 months	Unknown sex	https://happywhale.com/individual/25898
IBJ-4463			3 years 4 months	Unknown sex	https://happywhale.com/individual/35050
IBJ-2003			14 years 3 months	Unknown sex	https://happywhale.com/individual/34743
IBJ-3918			7 years 3 months	Unknown sex	https://happywhale.com/individual/33763
IBJ-0261			29 years 5 months	Unknown sex	https://happywhale.com/individual/32898
RSL-131			1 year 6 months	Unknown sex	https://happywhale.com/individual/44788
Raulzito Oleg			3 years 3 months	Male	https://happywhale.com/individual/45068
IBJ-1309			8 years 4 months	Male	https://happywhale.com/individual/19672
Nina			11 years 8 months	Female, recorded as a calf in 2004 and pregnant in 2016^47^	https://happywhale.com/individual/26347
IBJ-2907			8 years 1 months	Unknown sex	https://happywhale.com/individual/2945
IBJ-4636			3 years 6 months	Unknown sex	https://happywhale.com/individual/37484
PMC0003			2 years 6 months	Unknown sex	https://happywhale.com/individual/25687
PAN-1834			6 months	Unknown sex	https://happywhale.com/individual/36029
PAN-1055			6 years 7 months	Unknown sex *Note* Recorded in WAP only 6 months before it was recorded in South Orkney Island	https://happywhale.com/individual/34550

Credit is given for images used, with permission, when not owned by the authors and their institutions.

‘Δt’ is the shortest time interval (in years and months) between migratory destinations.

### Photoidentification and humpback whale sexual maturity age

These photo-identification records also revealed additional information about age of sexual maturity. For instance, the female named Nina was first photo-identified in her birth year in Abrolhos, Brazil, and then photo-identified pregnant 11 years later along the WAP (pregnancy determined by hormone analysis of a skin biopsy sample^47^). This time gap coincides with the mean age at first calving of humpback whales in Southeastern Alaska (11.8 years)^48^. However in this case we have no data to determine whether parturition was successful at the breeding area, nor whether this was the first pregnancy for this animal.

## Discussion

We found new evidence of summer co-occurrence of two recovering humpback whale breeding populations from the Atlantic (Brazil, BSA) and Pacific (Central and South America, BSG) in the West Antarctic Peninsula feeding area. The temporal pattern of resightings of BSA individuals (older sightings in South Georgia and South Sandwich Islands and more recent sightings off the WAP) suggests BSA whales may be making more extended feeding area movements in recent years. Photo-identifications collected from South Georgia and South Sandwich Islands (Table 1) add to the previous evidence that this area is a known feeding area for whales belonging to BSA. For example, the oldest record of an individual photo-identified at Abrolhos Bank, Brazil in 1989 and matched to its feeding area was for whale IBJ-0261 (Table 1). This individual was photo-identified almost 30 years later in the South Sandwich Islands in 2019, the feeding area for BSA. Photo-identifications available in Happywhale from the South Georgia and South Sandwich Islands mostly come from recent efforts and presently include only 322 identified individuals, 7% of the WAP sample size, thus this effort offers a limited sighting history for photo-identification matching, perhaps explaining the lower number of matches compared to the matches seen between the WAP and wintering grounds in Pacific Central and South America.

Available photo-identification data from the South Orkney Islands is also limited in the Happywhale dataset, but of 17 individually identified humpback whales, two individuals (PAN-1055 and PAN-1834) have been resighted on BSG breeding areas off Ecuador, Colombia, and Panama (Table 1, Figs. 1, 2). Notably, individual PAN-1055 may have migrated north late (or not at all) during the 2019 winter providing information on recent unusual movements around southern polar waters. The breeding destination of the other 15 identified whales in the South Orkney Islands remains to be determined.

Despite differences in photo-identification effort among breeding areas, this study reveals the first evidence of whales moving from Brazil into BSG feeding areas, reinforcing the proposed “Southern Ocean Exchange”. As such, the WAP feeding area is used by two different breeding stocks, BSG and BSA, which were previously strongly differentiated, and now co-occur in polar waters. Increasing photo-identification efforts in this region should provide further evidence of this assessment. The number of individuals that migrate between these two breeding stocks has been estimated to be 1–1.5 whales per season^33^. Two BSA individuals (named Nina and Raulzito Oleg) were photo-identified off the WAP, crossing the boundary between feeding areas attributed to BSA and BSG in the same period (January 2016, austral summer) and relatively close together (Fig. 1c). This is strong evidence that at least in recent years the use of BSG feeding areas by BSA whales is not a rare event.

While humpback whales are nearly global in their distribution, there is strong evidence that the equator represents a barrier to movement of this species^9,49^. Mitochondrial and nuclear genetic differentiation is stronger among humpback whales from different hemispheres (North Atlantic, North Pacific, and Southern Hemisphere) than among humpback whales from three Southern Hemisphere ocean basins (Indian, South Pacific, and South Atlantic), suggesting that gene flow is greater across the Southern Hemisphere oceans than across the equator^9,50^. This has led to a proposal to separate humpback whales into North Atlantic, North Pacific, and Southern Hemisphere subspecies^9,50^. Levels of genetic differentiation are weaker among Southern Hemisphere breeding stocks^12,13^ but sufficiently strong that they have been considered demographically independent populations, with population assessments of recovery from exploitation carried out at the level of each breeding stock^51^. Within the Southern Hemisphere, inter-population genetic differentiation may be anticipated to decrease, as populations recover from whaling and migratory movements between populations increase. If the crossing of individuals from BSA into BSG feeding areas is a recent event, likely motivated by population recovery, the observations presented in this study support this hypothesis.

Mixing of Southern Hemisphere humpback whales on high latitude feeding areas (the Southern Ocean Exchange), sometimes leading to movements between breeding stocks, might be influenced by geographic features such as landmasses, and oceanographic barriers such as major ocean currents that restrict movements. Additionally, El Niño and La Niña climatic events influence the distribution of prey and therefore may potentially influence the migratory destination and timing of migration^41^. The observed long-range movements between Southern Hemisphere oceans may also result from increasing population size^52^ and expanding distribution range, as whales colonize new areas and reoccupy historical breeding and feeding areas^5,53,54^.

Unique features that determine oceanographic and ecological characteristics of the Southern Ocean, such as climate and sea-ice extent variability that affect krill productivity and distribution dynamics, can also influence individual decision-making about migratory paths to these feeding destinations^55^. Observed shifts in migration timing of humpback whales from Antarctic feeding areas to earlier arrival at breeding areas in the Eastern Tropical Pacific off Colombia are also believed to be associated with population growth (e.g., high pregnancy rates detected in WAP^56^) and reductions in sea ice coverage and pack mass during the austral autumn, which, in turn, should influence krill availability in Antarctica^57^. When the autumn ice sheet has a larger mass, more krill can find food in winter^58,59^ and during the following summer, there are more prey available for whales. In years with larger autumn ice sheets, humpback whales arrive later to Colombian waters the following winter. Prey availability is probably used by whales as a cue to time their migration to wintering areas^57^.

Krill availability along the WAP correlates with reproductive rates in BSG whales, suggesting the importance of krill to the dynamics of this population^60^. Although krill abundance in the southwest Atlantic sector is one of the largest in the Southern Ocean, krill density in South Georgia is declining^61,62^ and show interannual fluctuation linked to climate variability^63^, with population-level impacts on other krill predators using South Georgia waters^64^. High krill density values (> 30 g m^−2^) are interspersed with years with low density (< 30 g m^−2^) with fluctuations every 4–5 years^65^. The relationship between BSA whale population growth (12% per year^66^) and krill availability is yet to be established but is assumed to be similar to that found for BSG. Increasing fluctuations in krill availability in the northern Scotia Arc may also be forcing humpback whales to venture further from their traditional feeding areas in order to feed. Melting sea ice around the WAP may open up new habitats and modulate krill densities and distributions across the Peninsula and into the Scotia Arc^67^ which, in turn, may have a dampening effect on whale population growth while increasing the likelihood that humpback whales from different populations will venture into areas that were not traditionally utilized for feeding in that part of the ocean.

Behavioural traits such as humpback whale song and its evolution and transmission are also facilitated by the Southern Ocean Exchange. In humpback whales, songs are produced by males^68,69^. Molecular data also suggests most movements between breeding grounds, and associated gene flow, may be male-driven^33^. Male carriers of a particular song may therefore introduce it to another population. Cultural transmission of song patterns by males has been shown to occur between west and east coasts of Australia^70^ and across the South Pacific Ocean^15^. The lower site fidelity of males therefore facilitates song sharing among breeding grounds in the Southern Hemisphere^16,71^. The co-occurrence of whales from different populations could also contribute to the spread of infectious diseases. In seabird populations, extensive winter mixing has been suggested as an important factor in disease transmission^72^. For example, morbillivirus was recently identified in the blow of humpback whales in Brazil^73^ and it could be introduced to other breeding areas through whales that migrate away from their natal breeding and feeding grounds.

Here we have considered how population growth, climate change and oceanic productivity may be acting synergistically to increase the porosity of boundaries among feeding areas of different humpback whale breeding stocks. Underscoring the Southern Ocean Exchange hypothesis of porous Southern Ocean boundaries for humpbacks are the international collaborations among academics and citizens which have made these observations possible. Traditionally, fluke comparisons between regions were very time-consuming as they were carried out manually by researchers visually inspecting all images^74^. New algorithms and tools for automated fluke matching, such as the one developed and implemented by Happywhale^43^ have enormously improved matching rates and also facilitate the detection of low-frequency movements. Photo-identification sample sizes used in humpback whale stock comparisons jumped 50-fold when citizens started to participate in biodiversity monitoring, facilitated by public campaigns, tourism, and the development of user-friendly platforms to engage and receive feedback on their contributions. In ours and other cases, these contributions add great complementary value to dedicated scientific research^75,76^. Global citizen engagement informs, at a much faster pace, stakeholders’ actions towards conservation of marine life. The relative cost of citizen science compared to more academic approaches in wildlife monitoring may change how conservation science is made and perceived by society in the near future.

## Methods

Digital images from research collaborator and citizen science contributors of the ventral surfaces of humpback whale tails (flukes) photographed in Central and South American breeding areas (~ 6476 photo-identified individuals in BSA and 3060 photo-identified in BSG) and feeding areas near South Georgia and the South Sandwich Islands (associated with BSA, n = 322 photo-identified individuals) and the WAP (associated with BSG, n = 4668) were uploaded to the web platform Happywhale (Table 1)^43^. The number of Brazilian individuals identified may be overestimated since the catalogues from Brazil are not fully reconciled (*i.e.*, there may be repeat sightings of individuals in this dataset) and are pending cross-validation on Happywhale. Uploaded images were matched via automated image recognition^43^ to a dataset of 47,122 known individual humpback whales worldwide, of which 4990 (10.6%) have been photo-identified in the feeding areas of BSG or BSA. Individual fluke matches are presented in Table 1.

## Data Availability

The matched images are publicly available under a Creative Commons Attribution-NonCommercial-ShareAlike 4.0 License (http://www.happywhale.com).

## Code availability

An open-source repository for the original algorithm, as posted at the completion of the Kaggle competition though which it was developed, is available via https://www.kaggle.com/c/humpback-whale-identification/discussion. Access to the implemented algorithm and supporting information architecture is available for use, at no cost, via the web platform www.happywhale.com.
